# Curiosity model policy optimization for robotic manipulator tracking control with input saturation in uncertain environment

**DOI:** 10.3389/fnbot.2024.1376215

**Published:** 2024-05-01

**Authors:** Tu Wang, Fujie Wang, Zhongye Xie, Feiyan Qin

**Affiliations:** ^1^College of Computer Science and Technology, Dongguan University of Technology, Dongguan, China; ^2^College of Outstanding Engineers, Dongguan University of Technology, Dongguan, China

**Keywords:** robotic manipulator, input saturation, uncertain environment, model-based reinforcement learning, intrinsic motivation, buffer schedule

## Abstract

In uncertain environments with robot input saturation, both model-based reinforcement learning (MBRL) and traditional controllers struggle to perform control tasks optimally. In this study, an algorithmic framework of Curiosity Model Policy Optimization (CMPO) is proposed by combining curiosity and model-based approach, where tracking errors are reduced via training agents on control gains for traditional model-free controllers. To begin with, a metric for judging positive and negative curiosity is proposed. Constrained optimization is employed to update the curiosity ratio, which improves the efficiency of agent training. Next, the novelty distance buffer ratio is defined to reduce bias between the environment and the model. Finally, CMPO is simulated with traditional controllers and baseline MBRL algorithms in the robotic environment designed with non-linear rewards. The experimental results illustrate that the algorithm achieves superior tracking performance and generalization capabilities.

## 1 Introduction

Robotic manipulator trajectory tracking control as a classical control task has been broadly discussed in academia and industry. Previous knowledge of the kinematic and dynamic model of the robotic manipulator is required by most traditional controllers (Thuruthel et al., 2019). Several estimation methods such as parameter identification (Zhang et al., 2024) and state estimation (Wei et al., 2023) have been proposed to alleviate the tolerance of the robot model. However, it is still essential to have knowledge of the fundamental model and recalibrate the parameters for various types of robotic manipulators (Íñigo Elguea-Aguinaco et al., 2023). Reinforcement Learning (RL) achieves maximum reward by training agents in an environment, without knowing the specific robot model. Model-Free Reinforcement Learning (MFRL) can accomplish these types of skills rather than just programming a fixed task through a procedure (Hu et al., 2020). Therefore, controller tuning time can be saved by using an agent to operate. The primary limitations lie in the high cost of training due to model-free methods, requiring extensive data and inefficient interaction with the real world (Luo et al., 2022).

The emerging model-based methods deliver higher sampling efficiency than model-free methods through learning a dynamic model (called the world model in this study) of the environment (Peng et al., 2018; Luo et al., 2022). Enormous amount of environmental simulation data is generated from the world model for agent training, which remarkably reduces the cost of data generation by interacting with real robotic manipulators (Hu et al., 2020). This advantage offers compelling potential for applications in many complex environments, such as robotic manipulators (Pane et al., 2019; Thuruthel et al., 2019; Lu et al., 2021). However, due to the robotic uncertainties, it is difficult to train the world model with limited prior knowledge. Furthermore, as elaborated in the study by Guo et al. (2021), another challenge for robot learning control may be input saturation and external disturbance, which are frequently encountered and unavoidable in mechanical systems. An effective way to mitigate these problems is to increase the agent's ability to explore. Well-optimized agents can discover the general shape of these challenges and provide control methods accordingly.

Intrinsic motivation maps novelty-based rewards via digging into implicit features of the environment to sweeten the efficiency of agent exploration in unknown environments (Sun et al., 2022b). Curiosity-driven, as its offshoot, evaluates the novelty of states through self-supervised learning, which is later used to compute intrinsic rewards (Burda et al., 2018a). This technique uses standalone modules that can be easily integrated into reinforcement learning frameworks. Hence, it has been widely discussed and applied to improve sampling efficiency (Sun et al., 2022b). Various applications have demonstrated the effectiveness of curiosity-driven approaches in both dense and sparse reward scenarios (Gao et al., 2023). Nevertheless, inappropriate ratio design can interfere with expressing extrinsic rewards in dense reward settings (Zhelo et al., 2018). Some references have explored more complex relevance (Wu et al., 2022) and contrastive learning (Sun et al., 2022a) to mitigate the instability of pure-state features. Unfortunately, these attempts have limited effectiveness in enhancing robot environments that only provide physical information. Curiosity-driven expression of intrinsic rewards can be augmented by using dynamically shifting ratios instead of irrationally fixed designs, which require a rational evaluation metric.

In this study, MBRL is adopted to strengthen the efficiency of agent training. Meanwhile, integrating intrinsic curiosity with world models is proposed as a scheme to elevate performance in uncertain environments with robot input saturation. Based on the above, the Curiosity Model Policy Optimization (CMPO) framework is proposed, which efficiently blends curiosity with the world model by adaptively adjusting the changes in intrinsic rewards and reward ratios through rich evaluation metrics. The agent is responsible for configuring the controller gain to provide the necessary inputs to the robotic manipulator in the environment.

The CMPO algorithmic framework offers the benefits of fast data collection and curiosity-driven exploration for world model. This means that agents trained using this framework can work alongside traditional controllers to significantly enhance the performance of robotic manipulators. The main work and contributions are summarized below:

- Unlike the approach in which intrinsic rewards are always defined as positive in the study by Pathak et al. (2017), a positive–negative intrinsic evaluation approach is defined, which adopts the world model to predict the effects of intrinsic rewards. Motivated by Haarnoja et al. (2019), by simply designing the intrinsic reward target, the adaptive ratio is proposed to be automatically tuned during curiosity exploration. These two modules work together to improve the sampling efficiency of the world model and agent.
- Inspired by the FVI bound theory (Lai et al., 2021) and the use of curiosity (Pathak et al., 2017), the data novelty distance is designed to adjust the ratio of data sampled from the environment buffer and model buffer in each training episode, reducing the influence of external disturbance. Additionally, a non-linear reward system is created to enhance agent training. Sensible data buffer scheduling and the use of reward systems increase the training speed of the agent.
- Building upon the foundation of MBPO (Janner et al., 2021), CMPO overcomes the obstacles of world model fitting in uncertain environments with robot input saturation. Training performance comparison exhibits superior control performance and generalization ability. Ablation experiments demonstrated the help provided by each module. Moreover, parameter sensitivity experiments provide valuable references for CMPO hyperparameter selection.

## 2 Related works

### 2.1 Model-based RL

Within the realm of MBRL, Dyna-Q-like methods (Peng et al., 2018) constitute a distinct category. Rather than relying on a single model, ME-TRPO (Kurutach et al., 2018) employs a B-length bootstrap model, which is trained in SLBO (Luo et al., 2021), utilizing a multi-step L2 loss function. During the same period, PETS (Chua et al., 2018) systematically interpreted the ensemble model as resolving aleatoric uncertainty. MBPO (Janner et al., 2021) exploits their advantages to effectively improve model sampling efficiency by proving monotonic lower bound guarantees for branch prediction. Subsequently, BMPO (Lai et al., 2020) further extends MBPO to bidirectional branching forecasts. AMPO (Shen et al., 2020) reduces the mismatch between the model and environment data. Nevertheless, frequent updates distort the predictions of the network and the appropriate start-stop scheme is not given in the study by Luo et al. (2022). In this study, MBPO is utilized in the CMPO to ensure monotonic bounds, and scheduling theory (Lai et al., 2021) is employed to ensure that training data can be sampled correctly.

### 2.2 RL with traditional controller

Combining reinforcement learning with controllers can facilitate task execution by exploiting their advantages simultaneously. By exporting the control gain for the traditional controller via RL, Wang et al. (2020) uses DQN to control the trajectory tracking of the mobile robot. Unlike the method of the study by Lu et al. (2021), the agent output is linearized with the controller output in the study by Xu et al. (2019) and a non-linear fuzzy reward system is designed for DDPG. Hu et al. (2020) further employs the RL approach with kernel model to elevate sampling efficiency and tracking capability. In this study, a similar view in the study by Xu et al. (2019) is utilized to design the non-linear rewards and follow the simulation experiment design methodology in the study by Hu et al. (2020).

### 2.3 Curiosity-driven exploration

Curiosity-driven exploration maps novelty-based intrinsic rewards by mining implicit features of the environment and the agent (Stadie et al., 2015). At the outset, Li et al. (2020) allocates rewards through static and dynamic encoders. Instead of focusing on individual states (Burda et al., 2018b), the approach in the study by Yang et al. (2019) evaluates intrinsic rewards by extracting characteristics of changes between states. It is worth noting that the ICM framework in the study by Pathak et al. (2017) concurrently employs forward and inverse dynamic encoding of state features, which significantly triggers intrinsic rewards for changes. By combining previous work, Huang et al. (2022) proposes a unified curiosity architecture. Recent research has focused on re-evaluating the novelty of states using novel methods such as context learning (Lee et al., 2020), contrastive learning (Huang et al., 2022; Sun et al., 2022a), and relevance (Grill et al., 2020; Wu et al., 2022). These approaches are unable to assist with robot physical states that lack redundant information. Therefore, the classical self-supervised exploration (Pathak et al., 2017; Li et al., 2020) is employed for state feature extraction and evaluation of curiosity in this study.

## 3 Problem description

In this section, the trajectory tracking problem is presented for an n-joint robotic manipulator. Consider a dynamic model for a robotic manipulator (Cao et al., 2021) operating in an uncertain environment:


(1) $$\begin{array}{l} M\left(q\right)\ddot{q}+C\left(q,\dot{q}\right)\dot{q}+G\left(q\right)=\tau \left(t\right)+d\left(t\right) \end{array}$$

where $q\in {\mathbb{R}}^{n},\;\overset{•}{q}\in {\mathbb{R}}^{n},$ and $\ddot{q}\in {\mathbb{R}}^{n}$ denote the joint angles, velocities, and accelerations, respectively; **τ**(*t*)∈ℝ^*n*^ denote the joint torques; ***d***(*t*)∈ℝ^*n*^ denote the external disturbance force. *M*(***q***)∈ℝ^*n*×*n*^ expresses the inertial matrix; $C\left(q,\overset{•}{q}\right)\in {\mathbb{R}}^{n\times n}$ represents the centrifugal-Coriolis matrix; *G*(***q***)∈ℝ^*n*^ is the gravity potential force, and each of them consists of known and unknown parts (Lu et al., 2021):


(2) $$\left\{ \begin{array}{l} M(q)=M_{0}(q)+M_{\Delta }(q)\\ C(q,\;\dot{q})=C_{0}(q,)+C_{\Delta }(q,\;\dot{q})\\ G(q)=G_{0}(q)+G_{\Delta }(q) \end{array}\right.$$

where (·)_0_ denotes the known part and (·)_Δ_ denotes the unknown part, which is caused by environmental variations or measurement errors. In the actual training environment, all the dynamic parameters are unknown to the agent.

Define ***x*_1_**(*t*) = ***q***(*t*), $x_{2}\left(t\right)=\overset{•}{q}\left(t\right)$. Subsisting (2) into (1) and then rewriting it with ***x*** gives:


(3) $$\begin{array}{l} \{\begin{array}{l} {\dot{x}}_{1}=x_{2}\\ {\dot{x}}_{2}=M_{0}^{-1}\tau +M^{-1}d+l \end{array} \end{array}$$

where $l\left(t\right)=M^{-1}\left[-C\left(q,\overset{•}{q}\right)\overset{•}{q}-G\left(q\right)\right]+{\bar{M}}_{\Delta }\tau$ is the uncertainty modeling depends on the system state, where ${\bar{M}}_{\Delta }=M^{-1}-M_{0}^{-1}$. The uncertainty ***l*** and the disturbance ***d*** are unknown and are assumed to be bounded (Guo et al., 2021).

Trajectory tracking errors can then be defined as follows:


(4) $$\begin{array}{l} \{\begin{array}{l} e_{1}=x_{1}-x_{d}\\ e_{2}={\dot{x}}_{1}-{\dot{x}}_{d}=x_{2}-{\dot{x}}_{d} \end{array} \end{array}$$

where ***x***_*d*_ is the desired trajectory, ***e***_1_ mean the tracking position error, and ***e***_2_ indicate the tracking velocity error. ***x***_1_, ***x***_*d*_, ***x***_2_, and **ẋ**_*d*_ are assumed to be bounded in the control system and can be observed precisely.

Consider an optimal control problem with finite time horizon length *N*. Given an initial state *s*_0_, the following minimum optimization problem is expected to solve (Brunke et al., 2022):


(5) $$\begin{array}{l} J^{\pi^{*}}(s_{0})=\;\underset{t_{0:\;N-1}}{\min}J(s_{t},u_{t})=\underset{t_{0:\;N-1}}{\min}\sum_{t}e_{1}(t)+u_{t} \end{array}$$

s.t. ***s***_t_ + 1 is derived recursively from Equation 3,


$$\begin{array}{l} u_{t}=\tau_{t}+d_{t} \end{array}$$

where ***u*** denotes the control input and is assumed to be bounded, i.e., $u\leq \bar{u}$, where **ū** is a known vector; π^⋆^ depicts the optimal policy. According to Equation 5, the objective is to design a controller that achieves the dynamic iteration process of Equation 3 by minimizing the sum of the tracking error ***e***_1_ and input cost ***u***.

## 4 Preliminaries

### 4.1 Reinforcement learning

As a continuous action space problem, robotic trajectory tracking control can be defined in a time-limited Markov decision process, which can be described by a quaternion set (S,A,P,R) (Mnih et al., 2013; Brunke et al., 2022; Kapturowski et al., 2022). The state space S and the action space A are continuous, and the policy π provides a probability of transition from current state $s_{t}\in S$ with current action $a_{t}\in A$ to next state $s_{t+1}\in S$: *p*(*s*_*t*+1_|*a*_*t*_, *s*_*t*_)= π(*a*_*t*_|*s*_*t*_) ∈P(A). The emits result $r\left(a_{t},s_{t}\right)\in R$ means the reward of each transit step, the sum of which denotes the reward of episodes. Our goal is to train a policy π to obtain the most expected rewards from every episode, which can be defined as follows:


(6) $$\begin{array}{l} \pi^{⋆}=\arg\underset{\pi }{\max}\underset{t}{\sum }{𝔼}_{\left(a_{t},s_{t}\right)\in \pi \left(a_{t}|s_{t}\right)}\left[r\left(a_{t},s_{t}\right)\right] \end{array}$$

where π^⋆^ denotes the optimal policy.

### 4.2 Soft actor critic

Soft Actor Critic (SAC) (Haarnoja et al., 2019) is an off-policy method based on the actor-critic algorithm. This approach uses the idea of maximum entropy to enhance the ability to explore policy:


(7) $$\begin{array}{l} \pi^{⋆}=\arg\underset{\pi }{\max}\sum {𝔼}_{\left({\text{s}}_{t},{\text{a}}_{t}\right)\sim \rho_{\pi }}\left[r\left({\text{s}}_{t},{\text{a}}_{t}\right)+\alpha H\left(\pi \left(\cdot |{\text{s}}_{t}\right)\right)\right] \end{array}$$

where ρ_π_ denotes the quaternion set of the Markov decision process. The actor network exports the action policy and updates its parameters θ^*a*^ using Equation 8.


(8) $$\begin{array}{l} L_{\theta^{a}}={𝔼}_{{\text{s}}_{t}\sim \rho_{\pi }}\left[{𝔼}_{{\text{a}}_{t}\sim \pi_{\theta^{a}}}\left[\alpha \log\left(\pi_{\theta^{a}}\left({\text{a}}_{t}|{\text{s}}_{t}\right)\right)-Q\left({\text{s}}_{t},{\text{a}}_{t}\right)\right]\right] \end{array}$$

where *Q*(**s**_*t*_, **a**_*t*_) denotes the critic network, which adopts the Equation 9 to update the parameter θ^*Q*^.


(9) $$\begin{array}{l} {ℒ}_{\theta^{Q}}=E_{(s_{t},a_{t})\sim \rho_{\pi }}[\frac{1}{2}(Q_{\theta^{Q}}(s_{t},a_{t})-(r(s_{t},a_{t})\\ \;+\;\gamma E_{s_{t+1}\sim \rho_{\pi }}[V_{\theta^{\hat{Q}}}(s_{t+1})]){)}^{2}] \end{array}$$

In practice, the target critic network $\hat{Q}\left({\text{s}}_{t+1},{\text{a}}_{t+1}\right)$ is used to approximate value network *V*(**s**_*t*+1_), which avoids overestimating the state value. Finally, the maximum entropy adaptive exploration of Equation 7 is achieved by the adaptive temperature coefficient α.


(10) $$\begin{array}{l} L_{\alpha }={𝔼}_{{\text{a}}_{t}\sim \rho_{\pi }}\left[-\alpha \log\pi_{\theta^{a}}\left({\text{a}}_{t}|{\text{s}}_{t}\right)-\alpha \bar{H}\right] \end{array}$$

where $\bar{H}$ is a lower bound on the entropy expectation. Once the network parameters have been updated, all target networks are soft updated.

### 4.3 Dyna-Q-like MBRL

To simulate the real world, consider a dynamic model $f_{\theta }:{\mathbb{R}}^{\left|S|+|A\right|}\mapsto {\mathbb{R}}^{\left|S\right|}$. For continuous states and actions, a probability distribution sampling method is proposed so that the world model can be output as a probabilistic form as follows:


(11) $$\begin{array}{l} {\tilde{f}}_{\theta }\left(s_{t+1}|a_{t},s_{t}\right)=\text{P}\left(s_{t+1}|a_{t},s_{t};\theta \right) \end{array}$$

The learning objective of $\tilde{f}$ is to fit the real-world model *f*^⋆^ and give unbias output *s*_*t*+1_, which is trained by using the dataset $D_{env}={\left\{a_{n},s_{n},s_{n+1}\right\}}_{n=1}^{N}$ of length *N*, collected from the environment (Chua et al., 2018; Kurutach et al., 2018).

### 4.4 Controller saturation

To ensure accurate trajectory tracking, it is essential to provide sufficient input assistance with the robotic manipulator's pose transitions. However, saturated inputs can occasionally result in actual inputs being smaller than desired values, leading to poor tracking performance. Consequently, devising a controller that prevents such occurrences become an important issue. Suppose the joint torque **τ** is the only control input, then the input saturation can be described as follows:


(12) $$\tau_{i}=\{\begin{array}{ll} \tau_{\max} & ,\text{ if }\tau_{i}\geq \tau_{\max}\\ \tau_{i} & ,\text{ if }\tau_{\min}<\tau_{i}<\tau_{\max}\\ \tau_{\min} & ,\text{ if }\tau_{i}\leq \tau_{\min} \end{array},i=1,2,...,n$$

where τ_max_ is the maximum of torque and τ_min_ is the minimum of torque.

## 5 CMPO framework design

### 5.1 Architecture summary

**Figure 1:**
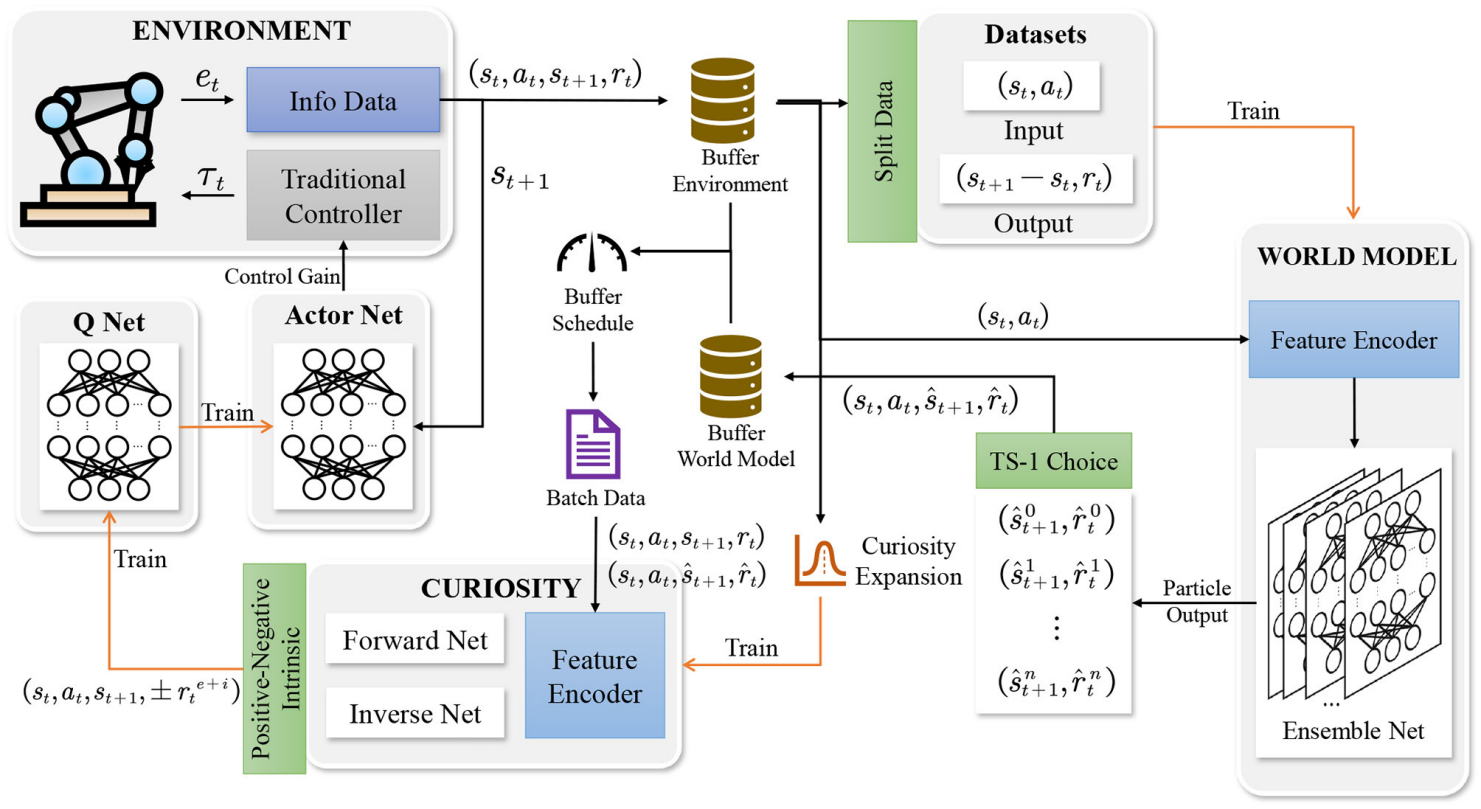
Schematic of the CMPO architecture. The agent interacts with the environment to store environment buffer data. The world model selects particles in the ensemble network to store generated model buffer data. In addition, the environment buffer data are used to train the curiosity network. Ultimately, the agent's actor network and Q network are trained using the buffer data evaluated via curiosity.

The CMPO framework contains an environment, a world model, an agent, and reply buffers. The control gain derived by the agent is input to the environment. The robotic manipulator will solve the dynamics based on the input and eventually output error and state information from the environment, which will stored in the environment buffer. Next, the environment buffer data are used to train both the world model and the curiosity network. Before training the world model, the environment buffer data are divided into training and evaluation datasets. After training the world model and the curiosity network, the world model generates simulation data and stores it in the world model buffer. Finally, the data from the environment buffer and model buffer are uniformly collected by the buffer scheduler, which is used to train the agent's actor network and Q network. The agent will continuously follow this loop to interact with the environment and train until convergence, and more detailed frameworks are shown in Figure 1. Specific implementation details are shown in Algorithm 1.

### 5.2 MBRL design

The MBPO technique supplements the branch prediction and ensemble model based on the Dyna-Q-like MBRL method, which increases the model sampling efficiency and training speed (Janner et al., 2021). Therefore, MBPO is used to design the world model under the CMPO algorithm. To better express and generalize the complex dynamic environment in a continuous Markov process, the Gaussian probabilistic neural network models are used to fit the environment to cope with the aleatoric uncertainty (Chua et al., 2018). Departing from Equation 11, the world model will predict the next state and reward. Thus, the model can be rewritten as follows:


(13) $$\begin{array}{l} {\tilde{f}}_{\theta }({\hat{s}}_{t+1},{\hat{r}}_{t}|a_{t},s_{t})=\text{P}({\hat{s}}_{t+1},{\hat{r}}_{t}|a_{t},s_{t};\theta )\\ \;=N\left(\mu_{\theta }\left(s_{t},a_{t}\right),\Sigma_{\theta }\left(s_{t},a_{t}\right)\right) \end{array}$$

where N(·) denote the gaussian distribution.

Since the states in the actual environment are less dimensional, the feature network in curiosity is used along with the model to encode its states, augmenting the feature extraction capability. Given that the environment changes slightly from step to step, the deterministic network (Luo et al., 2022) is applied to describe the predicted output of the world model. With the combination of the above changes, Equation 13 can be rewritten to satisfy the iterative output of the next state in Equation 5:


(14) $$\begin{array}{l} \left({\hat{s}}_{t+1},{\hat{r}}_{t}\right)=\left(s_{t+1},r_{t}\right)+{\tilde{f}}_{\theta }(\Delta {\hat{s}}_{t+1},\Delta {\hat{r}}_{t}|a_{t},s_{t})\\ \;=\left(s_{t+1},r_{t}\right)+N(\mu_{\theta }\left(\varphi (s_{t}),a_{t}\right),\Sigma_{\theta }(\varphi (s_{t}),a_{t})) \end{array}$$

where φ(·) denote the feature network and Δ(·) means the magnitude of change.

Using the basis of Equation 14 as the particle, the B-length bootstrap ensemble model $\tilde{f}=\left\{{\tilde{f}}_{\theta }^{1},{\tilde{f}}_{\theta }^{2},\cdots \;,{\tilde{f}}_{\theta }^{B}\right\}$ is adopted as the final world model, and then, a sum of negative log-likelihood loss is used as follows (Chua et al., 2018) :


(15) $$\begin{array}{l} {ℒ}_{\theta }=\sum_{t=1}^{N}{\left[\mu_{\theta }\left(\varphi \left(s_{t}\right),a_{t}\right)-s_{t+1}\right]}^{\text{T}}\cdot \Sigma_{\theta }^{-1}\left(\varphi \left(s_{t}\right),a_{t}\right)\\ \;\cdot \;\left[\mu_{\theta }\left(\varphi \left(s_{t}\right),a_{t}\right)-s_{t+1}\right]+\log\det\Sigma_{\theta }\left(\varphi \left(s_{t}\right),a_{t}\right) \end{array}$$

In practice, the output is obtained randomly from a particle by designing short trajectory sampling frequencies (TS1 method; Chua et al., 2018 ). At every environmental timestep, the TS-1 method selects a new particle ${\tilde{f}}_{\theta }^{i}$ from the ensemble model $\tilde{f}$ to serve as the branching prediction output for the next timestep. Branch rollouts are used to recursively generate new data from the world model by means of Equation 14 and store it in world model buffer $D_{model}$. Theory (Shen et al., 2020) suggests that incremental branch lengths can ensure advancements in real training, and the model returns are increased enough to guarantee the progression of base returns. For the agent, the SAC (Haarnoja et al., 2019) algorithm is used to optimize the policy with data collected from mixed $D_{env}$ and $D_{model}$.

### 5.3 Curiosity model design

#### 5.3.1 Self-supervised exploration

The curiosity network provides the agent with additional intrinsic rewards to overcome the uncertainty of the environment through more exploration, whereas the networks look for potential patterns in the environment through self-supervised learning. The self-supervised exploration of curiosity is inspired by the earlier structure (Pathak et al., 2017), which consists of a forward network and an inverse network. Due to the simplicity of the robot information, the states are encoded by the same feature networks before being fed into them.

The inverse network takes in the current and next state as inputs and outputs the current action. This allows the inverse network to learn how to derive the correct control gain. The loss function of the inverse network can be expressed as the mean squared error between the predicted and actual action (Pathak et al., 2017):


(16) $$\begin{array}{l} L_{\text{Inverse}}=\frac{1}{B}\overset{B}{\underset{n=1}{\sum }}{\left(a_{n}-{\hat{a}}_{n}\right)}^{2} \end{array}$$

where *B* is the batch size of each train step. The main task of the inverse network is to learn potential feature encodings so that they provide more feature semantics in the inputs for both the world model and the forward network.

The input of the forward network is identical to the world model, which uses a residual network (Li et al., 2020) to predict the feature encoding of the next state $\hat{\varphi }\left(s_{t+1}\right)$. The disparity between the predicted and actual encodings is used to measure curiosity and is defined as the loss function of the forward network:


(17) $$\begin{array}{l} L_{\text{Forward}}=r^{o}=||\hat{\varphi }\left(s_{t+1}\right)-\varphi \left(s_{t+1}\right)|{|}_{2}^{2} \end{array}$$

where *r*^*o*^ is the output intrinsic reward, as measured by coded differences. Combining Equations 16, 17 mentions in the study by Pathak et al. (2017):


(18) $$\begin{array}{l} L_{\text{Curiosity}}=\beta L_{\text{Forward}}+\left(1-\beta \right)L_{\text{Inverse}} \end{array}$$

where 1≥β≥0 is a scalar to balance $L_{\text{Forward}}$ and $L_{\text{Inverse}}$. Curiosity encourages the agent to look for new states (Li et al., 2020), which improves the agent's sampling efficiency. However, in uncertain environments, pessimistic incentives can lead robots to undertake risky actions. Hence, providing a method to evaluate intrinsic rewards plays a crucial role. The next part of this study provides a valuation and conversion strategy for intrinsic rewards.

#### 5.3.2 Positive–negative intrinsic

To further upgrade the sampling efficiency in uncertain environments with input saturation, an approach is proposed to strengthen the expression of curiosity, which is distinguished as positive and negative.

Let *X* denotes the sample of quaternion corresponding to the Markov process, and its two subscripts (·)_*e*_ and (·)_*m*_ denote the samples of the quaternion in $D_{\text{env}}$ and $D_{\text{model}}$, respectively, then *F* denotes the distribution function of these two datasets. Assuming that the trained world model is plausible according to the theory by Janner et al. (2021). Given the inputs in $D_{\text{env}}$, the model output distribution will also follow the distribution pattern in $D_{\text{env}}$. It can be further deduced that the generated dataset $D_{\text{model}}$ should exhibit similarity to the distribution of $D_{\text{env}}$ as follows:


(19) $$\begin{array}{l} F_{e}\left(X_{e}\right)\approx F_{m}\left(X_{m}\right) \end{array}$$

After training in $D_{\text{env}}$, data input in $D_{\text{env}}$ will produce small intrinsic rewards based on the convergence law of curiosity. Concerning $D_{\text{env}}$ and $D_{\text{model}}$, it is known from Equation 19 that curiosity still produces little reward when the world model produces the same distribution of data inputs. Based on the above, the current world model is ventured to use as a baseline for measuring curiosity.

**Figure 2:**
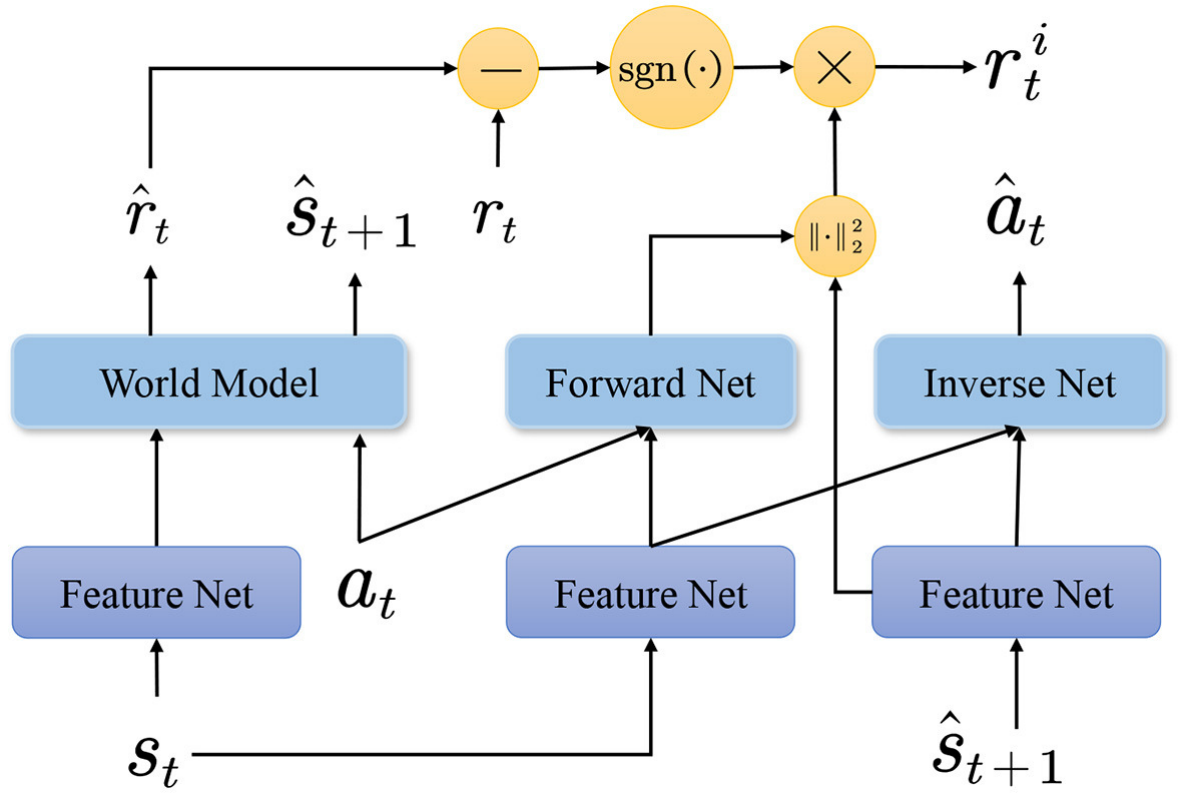
Flowchart of the algorithm for the curiosity model. The world model outputs predicted current rewards and predicted next states after inputting feature codes and actions for the current state. Meanwhile, the forward and inverse networks output the predicted current state features and current actions, respectively. The sign of the difference between the predicted reward and the current reward is referred to as the assessment of curiosity. The predicted features along with the actual features computed as intrinsic rewards are finally used as current intrinsic rewards along with the evaluation results.

$D_{\text{env}}$ becomes $D_{\text{env}}^{\prime }$ after collecting new data and no longer satisfies the Equation 19. Assuming that the distribution of $D_{\text{model}}$ follows the principle of being nearest and most similar to that of $D_{\text{env}}^{\prime }$. The predictive rewards of the world model are designed as a baseline so that the curiosity of $D_{\text{model}}$ is defined as positive. $D_{\text{env}}^{\prime }$ compares actual and predicted reward differences to assess whether curiosity is positive or negative. Taken together, the output positive–negative intrinsic reward can be rewritten as follows:


(20) $$r^{o}=\{\begin{array}{ll} \mathrm{sgn}\left(r_{t}-{\hat{r}}_{t}\right)\cdot {\left\|\hat{\varphi }\left(s_{t+1}\right)-\varphi \left(s_{t+1}\right)\right\|}_{2}^{2}, & \text{if }\left(r_{t},s_{t+1}\right)\in D_{\text{env }}^{\prime }\\ +1\cdot {\left\|\hat{\varphi }\left(s_{t+1}\right)-\varphi \left(s_{t+1}\right)\right\|}_{2}^{2}, & \text{if }\left(r_{t},s_{t+1}\right)\in D_{\text{model }} \end{array}$$

where sgn(·) denote the signal function. In practice, the sign is used instead of the difference to account for the mismatch between the model and the environmental data (Shen et al., 2020). Empirical evidence reveals that the sign is adequately robust to information bias. The specific flow of the algorithm is shown in Figure 2. Intrinsic rewards serve as appraisals of the agent's exploration process and, together with extrinsic rewards for environmental interactions, constitute rewards for the actual output. However, a terrible proportion of intrinsic rewards in an intensive reward environment can lead to the nullification of extrinsic rewards. In the next section, a method for adjusting the amount of intrinsic and extrinsic rewards is developed.

Remark 1. Curiosity is sensitive to state changes due to its state novelty design (Burda et al., 2018a). Research (Brunke et al., 2022) suggests that curiosity can have a negative effect when bad states are received. Equation 20 dynamically adjusts intrinsic rewards based on the quality of the state. When the environmental state is unfavorable, pessimistic curiosity hinders the exploration of the agent in that direction. Compared with the single method of evaluating state differences in the study by Pathak et al. (2017) and Burda et al. (2018b), positive–negative intrinsic helps the agent explore in a relatively better direction which improves the model sampling efficiency.

Remark 2. In the statement above, the nearest-similarity principle refers to a reasonable assumption that the input data generated by the next episode are also available in $D_{\text{model}}$ and have been used to train the agent recently. The reasonableness of the assumption is based on the phenomenon that each agent training samples a much higher proportion of model data than environment data. Moreover, the world model extrapolates predictions from the initial states within $D_{\text{env}}$, underlining the similarity between the distribution of $D_{\text{env}}^{\prime }$ and $D_{\text{model}}$.

#### 5.3.3 Curiosity expansion

The ICM (Pathak et al., 2017) moderates the impact of agent curiosity exploration using a fixed intrinsic reward ratio and is subsequently followed study by Burda et al. (2018b) and Yang et al. (2019). Nonetheless, the intrinsic reward decreases significantly with agent updates. It is difficult to tune an appropriate ratio for intrinsic reward. Instead of utilizing the fixed ratio, the acquisition of intrinsic rewards is treated as a constrained problem, where the mean value of intrinsic rewards is constrained, allowing intrinsic rewards to change adaptively during training. A similar approach is mentioned in the study by Boyd and Vandenberghe (2004) and Haarnoja et al. (2019), where it is applied to adaptively constrain the temperature coefficient in maximum entropy optimization. However, the curiosity-agent complexity association makes the optimization problematic.

In Equation 19, the relationship between the model data and the environment data has been mentioned, which has been shown the world model boosting for the agent. Thus, the maximum return of rewards from the agent in the intrinsic reward ratio constraint problem is equivalent to solving for the optimal fit of the curiosity and world model to the environment. Formally, the following constrained optimization problem is concerned:


(21) $$\begin{array}{l} \underset{{\;}_{{\tilde{f}}_{b_{0\;}:\;b_{T}}}}{\min}E_{(s_{t},s_{t+1},a_{t})\sim D_{\text{env}}}\left[\hat{\varphi }(s_{t+1})-\varphi (s_{t+1})\right]\\ \;+E_{(s_{t},a_{t})\sim D_{\text{env}}}\left[\tilde{f}(s_{t},a_{t})-f^{⋆}(s_{t},a_{t})\right]\\ \text{ s.t.}E_{(s_{t},s_{t+1},a_{t})\sim D_{\text{env}}}\left[r^{o}(s_{t},s_{t+1},a_{t})\right]\geq \bar{\text{R}} \end{array}$$

where $\bar{\text{R}}$ is the lower bound of target intrinsic reward and *b*_(·)_ denotes the training batch size index. There is no need to impose a constrained upper bound, as the output intrinsically rewards decreasing convergence during world model training. Similar to the method of Haarnoja et al. (2019), an iterative scheme is used to optimize from the last batch, modifying the constrained optimization of Equation 21 to minimize its dual problem as follows:


(22) $$\begin{array}{l} \underset{\eta_{b_{T}}\geq 0}{\max}\underset{{\;}_{{\tilde{f}}_{b_{T}}}}{\min}E_{\left(s_{t},a_{t}\right)\sim D_{\text{env }}}\left[\tilde{f}\left(s_{t},a_{t}\right)-f^{⋆}\left(s_{t},a_{t}\right)\right]\\ \;+\;E_{\left(s_{t},s_{t+1},a_{t}\right)\sim D_{\text{env}}}[\left(1-\eta_{b_{T}}\right)\cdot r^{o}\left(s_{t},s_{t+1},a_{t}\right)]+\eta_{b_{T}}\bar{\text{R}} \end{array}$$

where η_*b*_*T*__ is the dual variable. The variable η_*b*_*T*__ to be optimized in Equation 22 corresponds to the ratio variable when the world model is trained to fit the environment. The optimal dual variables are addressed as follows:


(23) $$\begin{array}{l} \begin{array}{cc} \eta_{b_{T}}^{⋆}=\arg\underset{\eta }{\max} & {𝔼}_{\left(s_{t},s_{t+1},a_{t}\right)\sim D_{\text{env}}}\left[\left(1-\eta \right)\cdot r^{o}\left(s_{t},s_{t+1},a_{t}\right)+\eta \bar{\text{R}}\right] \end{array} \end{array}$$

An iterative approach is adopted to batch optimization for $\eta_{b_{T}}^{⋆}$, since approximate optimization using neural networks is still valid. Equation 23 is rewritten as the optimized minimum loss function to facilitate consistent formatting:


(24) $$\begin{array}{l} L_{\eta }=\left(\eta -1\right)r^{o}-\eta \bar{\text{R}} \end{array}$$

The ratio optimized by $\eta_{b_{T}}^{⋆}$ may be lower than the manually designed one at the beginning. Nevertheless, as curiosity and ratio adapt competition converge, this method will still vary in the later steps, providing a boost for the agent. The detailed variation process is shown in Figure 7D.

After using the adaptive ratio variable, intrinsic reward *r*^*i*^ and total reward *r* are defined as follows:


(25) $$\begin{array}{l} \begin{array}{c} r^{i}=\eta \cdot r^{o}\\ r=r^{e}+r^{i} \end{array} \end{array}$$

where *r*^*e*^ is the extrinsic reward that can be obtained from the environment. Substituting this new reward *r*, the critic network update Equation 9 of SAC is made better. In addition, the critic network also allows for better evaluation and training of the actor network, increasing the overall training speed of the agent.

Remark 3. Differing from the previous intrinsic reward design (Pathak et al., 2017), *r*^*i*^ in Equation 25 has a lower bound $\bar{\text{R}}$. With the gradient optimization method, the ratio of intrinsic rewards is updated along with the curiosity network to ensure a pessimistic lower bound on intrinsic rewards. Furthermore, *r*^*i*^ converges toward reduction as indicated by the Equation 17. Through the interaction of decreasing convergence and expansive updates, intrinsic rewards remain influential despite the later stages of agent training. Subsequent experiments demonstrated the ability of the ratios to have a sustained impact, as shown in Figure 7D.

#### 5.3.4 Adaptive buffer schedule

Previous model optimization theories (Janner et al., 2021; Luo et al., 2021) provide a reliable basis for branch length enhancements, but they lack a method for selecting the buffer ratio. The buffer ratio helps the agent to sample a quota of environment data and model data, which are used for agent training. The environmental data are accurate, but the total amount of it is much less than the model data, which is not enough to train the agent. Conversely, the model data are sufficient, yet the insufficiently trained world model generates fatally biased data, leading to difficulties in convergence of agent training. The recent FVI bounds theory (Lai et al., 2021) provides the missing ratio theory, showing that a dynamically increasing ratio of environment buffer is beneficial for agent augment. Inspired by this remark, the specific method for judging the ratio is provided under the curiosity model.

**Algorithm 1:**
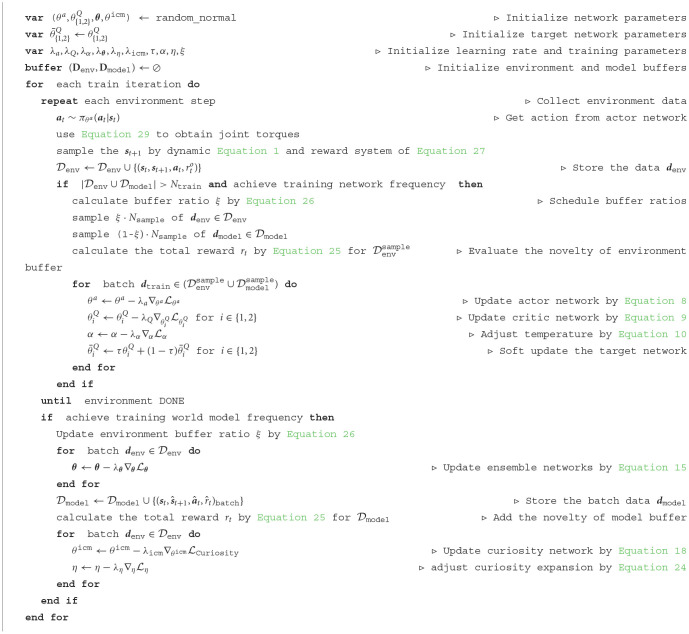
CMPO training algorithm.

The curiosity that converged for $D_{\text{env}}$ before training may be different for $D_{\text{env}}^{\prime }$, and thus, novelty ratio ξ is defined as follows:


(26) $$\begin{array}{l} \xi =\mathrm{clip}(\exp-(\sum_{D_{\text{env }}}\left|r^{o}\left(s_{t},s_{t+1},a_{t}\right)\right|\\ \;-\sum_{D_{\text{model }}}r^{o}\left(s_{t},{\hat{s}}_{t+1},a_{t}\right){)}^{2},\xi_{\min},\xi_{\max}) \end{array}$$

where clip(·) denotes the clip function, its first parameter is the raw value, which will clip to the lower bound value ξ_min_ and the upper bound value ξ_max_. The ratio ξ is defined as the proportion of data sampled from $D_{\text{env}}$ in each agent training step.

Remark 4. Effectively organizing environment and model buffer data is a challenging endeavor (Lai et al., 2021). Although the relevant theory (Lai et al., 2021) proves the importance of scheduling and provides a method for calculating the ratio, it uses an additional agent implementation that requires additional training, resulting in high implementation costs. The novelty distance uses known world models and buffer data for calculation, without requiring additional computational costs. In addition, the corresponding experimental ratio changes exhibit similarity to the theory, as shown in Figure 7C.

Remark 5. At the beginning of the training iterations, the agent will continue to explore novel data, and the difference in Equation 26 will be amplified, hence the ratio will be at its minimum value. As the number of iterations increases and the curiosity model learns more data, the agent gradually encounters less novel data, and accordingly the difference in Equation 26 is scaled down so that the ratio gradually increases to the maximum value. More detailed trends are shown in Figure 7C. This ratio, which reduces the bias of the world model toward the agent, is in line with this theory's main thrust.

## 6 Controller design

Curiosity model can help the agent to solve complex dynamic problems, but in practice, further assurance is essential that the agent will explore the robotic manipulator in safety (Brunke et al., 2022), which happens to be the strength of traditional controllers. PID is a simple model-free controller that can accomplish trajectory tracking tasks by giving suitable parameters (Wang et al., 2020). In this section, the CMPO is combined with PID controllers to provide suitable control gains to make the controllers achieve performance even in uncertain environments with input saturation.

### 6.1 Reward design

According to the definition of the dynamic equations shown in Equation 3, it is known that updating the system is related to position and velocity (Hu et al., 2020). Hence, non-linear rewards are designed for the positional factors, while the auxiliary speed factors use linear rewards, which are designed as follows:


(27) $$\begin{array}{l} r^{e}=\left(1-\frac{2}{\exp\left(-\varsigma \cdot \left(e_{1}-\sigma \right)\right)}\right)+e_{2}^{\text{T}}Ae_{2} \end{array}$$

where **σ** denotes the benefit threshold, ς>0 denotes the sensitive scale, and *A* is a semi-positive definite constant matrix that denotes the weight of velocity in extrinsic reward. σ is used to indicate the limit of positive and negative rewards, allowing the agent's capability to be as good as possible for that bound. To allow the agent to have a fast exploratory ramp-up period in rewards, ς can set the ratio of increase so that the agent can obtain rewards quickly after a certain level of performance is achieved.

### 6.2 Tracking controller design

The general definition of PID controller is as follows (Xu et al., 2019):


(28) $$\tau (t)=K_{p}e(t)+K_{i}\int_{0}^{T}e(t)dt+K_{d}\frac{d}{dt}e(t)$$

Discretizing Equation 28 and applying it to the robotic manipulator environment, it can be rewritten as follows:


(29) $$\begin{array}{l} \begin{array}{cc} \tau \left(t\right)= & K_{p}e_{1}\left(t\right)+K_{i}\left(\delta \cdot e_{1}\left(t-1\right)+\left(1-\delta \right)\cdot e_{1}\left(t\right)\right)+K_{d}e_{2}\left(t\right) \end{array} \end{array}$$

where *K*_*p*_, *K*_*i*_, and $K_{d}\in {\mathbb{R}}^{n}$ denote the proportional, integral, and differential gains of the n-joint dimension, respectively, and the integral term is approximated by proportional smoothing, which has a proportional value δ that denotes the memory of past errors. Equation 29 is used as a traditional control, which becomes the link between the action inputs and the conversion of the inputs from the robotic manipulator system.

The **τ** and ***d*** generated by the environment are iterated according to Equation 5, and new environment data are generated as a means of cyclic execution in the environment. In the CMPO framework, the environment steps are completed, and data are collected by interacting with the environment, inputting the current state *s*_*t*_ = {***x***, ***x***_*d*_, **ẋ**, **ẋ**_*d*_} to the agent and obtaining the action *a*_*t*_ = {*K*_*p*_, *K*_*i*_, *K*_*d*_} as controller gain input to the traditional controller.

**Figure 3:**
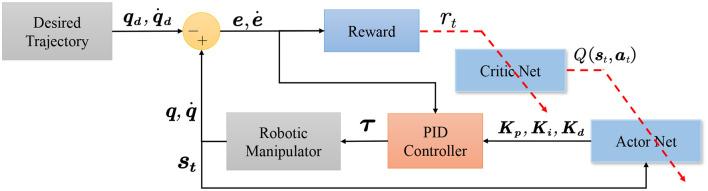
Schematic diagram of how robotic manipulator control works with reinforcement learning combined with a conventional controller. The PID controller calculates the joint torque based on the control gain of the actor network. Next, the robotic manipulator receives the torque and obtains the next state information. This information is used to calculate the next control gain and the error from the actual trajectory position. The input error is then utilized by the reward system to update the critic network, which, in turn, updates the actor network.

After the PID has obtained the controller gain, the torque is calculated based on the error input serves as the input of the force at each joint of the robotic manipulator. The robot manipulator calculates the position for the next step, which is used to determine the next state and the new error. Finally, the reward system gives an evaluation and updates the critic network and actor network sequentially. Figure 3 contains specific details regarding the control of environmental and updating cycles.

More details of the parameter update are shown in Figures 1, 3. Once the environment and model buffers have accumulated enough data, the agent will be updated in steps. Similarly, the world model is updated in episodes with all the data from the environment buffer. These two processes make up the update cycle of the controller. The pseudocode for the CMPO controller is shown in Algorithm 1.

## 7 Experimental results and analysis

### 7.1 Environmental configurations

Based on the scheme and methodology shown in the study by Hu et al. (2020), a simulation environment is set up for the tracking control of a two-link (2-DOF) manipulator in an uncertain environment with robot input saturation. The format of the parameters, inertial matrix, centrifugal and Coriolis force matrix, gravitational force effect of the robot, and their internal specific parameters are shown in Appendix A.

The control performance experiments of CMPO is compared with cutting-edge controllers. Advanced controllers not only use robotic models to improve control accuracy but also counteract environmental uncertainties through a sliding mode robust approach (Islam and Liu, 2011; Chertopolokhov et al., 2023). Unlike model-free controllers, model-based controller performance relies on the accuracy of the robotic model. In uncertain environments, robotic arm models potentially exist errors. Thus, different robotic model errors are employed in advanced controllers to compare the control performance with the CMPO algorithm.

In the environment, the individual states of the parameters of the robotic manipulator will be initially set as *q*_1_(0) = *q*_2_(0) = −0.5 and ${\overset{•}{q}}_{1}\left(0\right)={\overset{•}{q}}_{2}\left(0\right)=0.0$. The curves required to be tracked are designed as *q*_*d*1_(*t*) = sin(*t*) and *q*_*d*2_(*t*) = cos(*t*), thereby the tracking velocity is designed as ${\overset{•}{q}}_{d1}\left(t\right)=\cos\left(t\right)$ and ${\overset{•}{q}}_{d2}\left(t\right)=-\sin\left(t\right)$. Then, the extrinsic reward is set as σ = 0.35, ς = 2.0, and *A* = **0**, and the parameter of the PID controller is set as δ = 0.5. The step size is limited to 5, 000 for each episode of the environment, and the time variance of each step is limited to 0.01 s. Each episode of the environment simulates real-time information about the robot's trajectory for 50 s in agent training and the same step gap for 30 s in checkpoint simulation.

Depending on the experiment, the environments are categorized into three types, which are: basic, small-change, and large-change environments. Each of them adds saturation and disturbance, which are set as **τ**_max_ = 60, **τ**_min_ = −60, and |***d***| ≤ **2**. Each of the three settings adds saturation and disturbance, with disturbance being uniform noise occurring 75% of the time. For more specific settings of the robot parameters in each environment, refer to Appendix A.

### 7.2 Evaluation of algorithm

#### 7.2.1 Generalization ability

In this experiment, the performance of CMPO is compared with traditional PID (Wang et al., 2020) controller.

**Figure 4:**
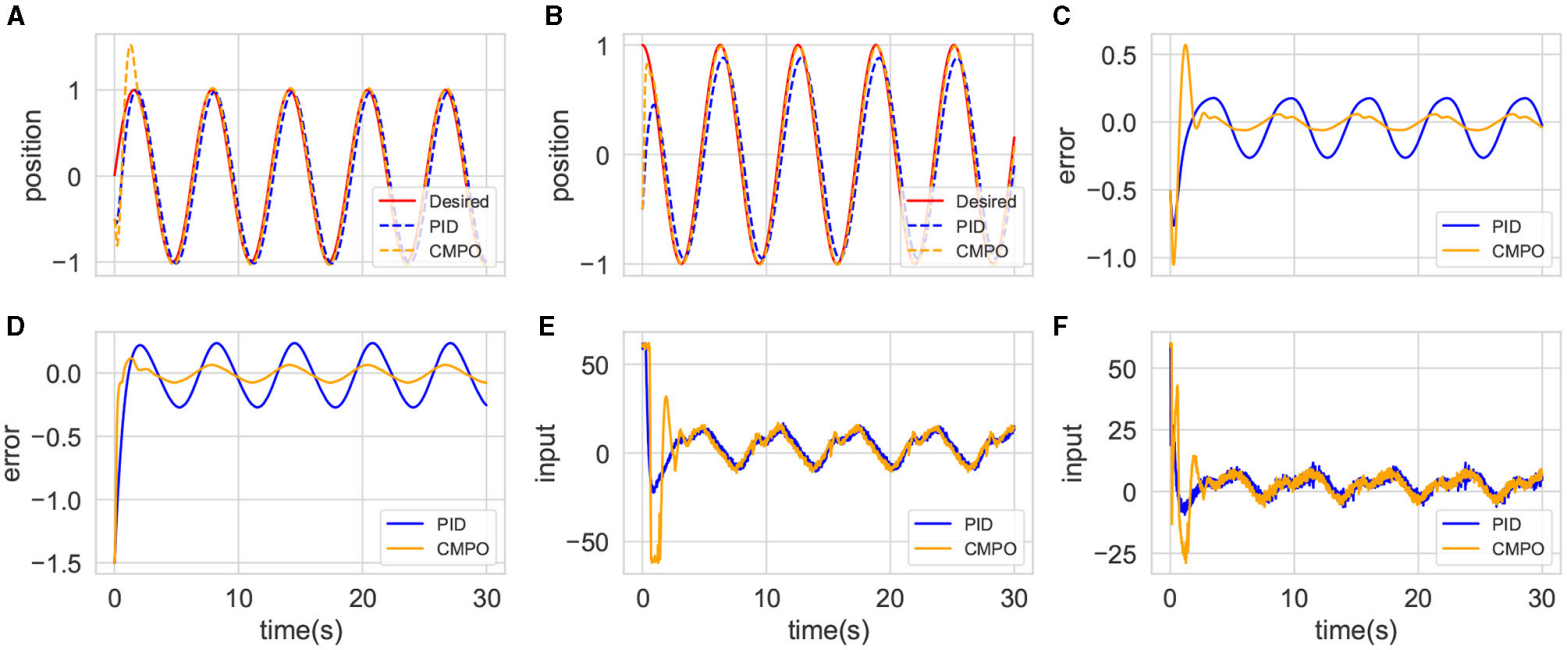
Comparison of tracking performance between CMPO and conventional PID controllers in the basic environment. **(A, B)** represent the curves of the position tracking over time for joint 0 and joint 1; **(C, D)** represent the curves of the tracking error over time for joint 0 and joint 1; **(E, F)** represent the curves of the input torque magnitude over time for joint 0 and joint 1.

The trained CMPO and the completed parameter-tuning PID algorithms are first simulated in the basic environment. The results are presented in Figure 4. It can be observed from Figures 4A, B that agent boosts the speed of the convergence afterward by sacrificing the performance of link 1 at the beginning. With this policy, it is obvious from Figures 4C, D that the converged tracking error CMPO is significantly better than that of PID.

**Figure 5:**
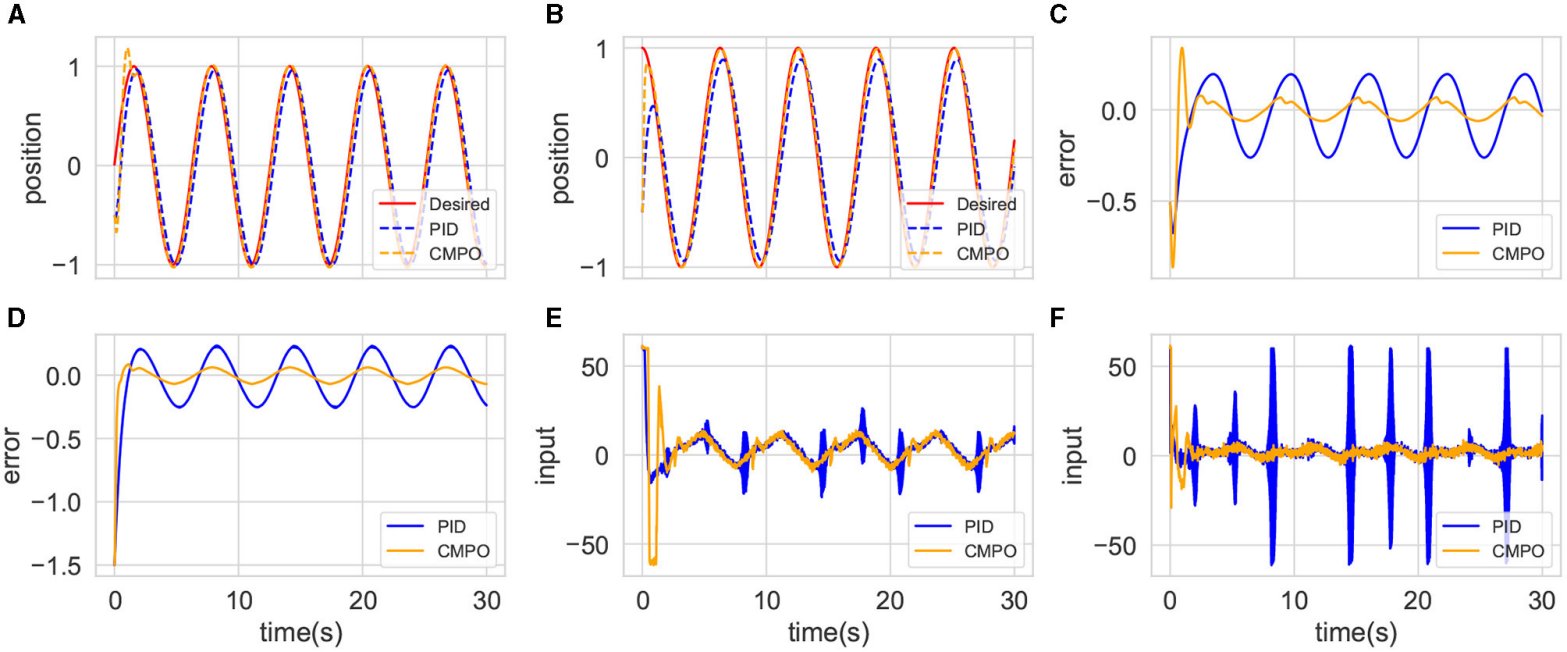
Comparison of tracking performance between CMPO and conventional PID controllers in the small-change environment with the same parameters in basic environment. The display content of each component image is similar to that of Figure 4.

The same models and parameters are then applied to simulate the small-change environment. This experiment compares their generalization abilities in a robotic environment with high tolerance. The results are shown in Figure 5. The tracking trajectories and errors of Figures 5A–D in this environment are similar to those of the basic environment, demonstrating the admirable generalization capabilities of traditional controllers. However, the input costs are shown to be different in Figures 5E, F, with CMPO having lower input than PID. This comparison empirically suggests that the agent input policy further enhances the control performance.

**Figure 6:**
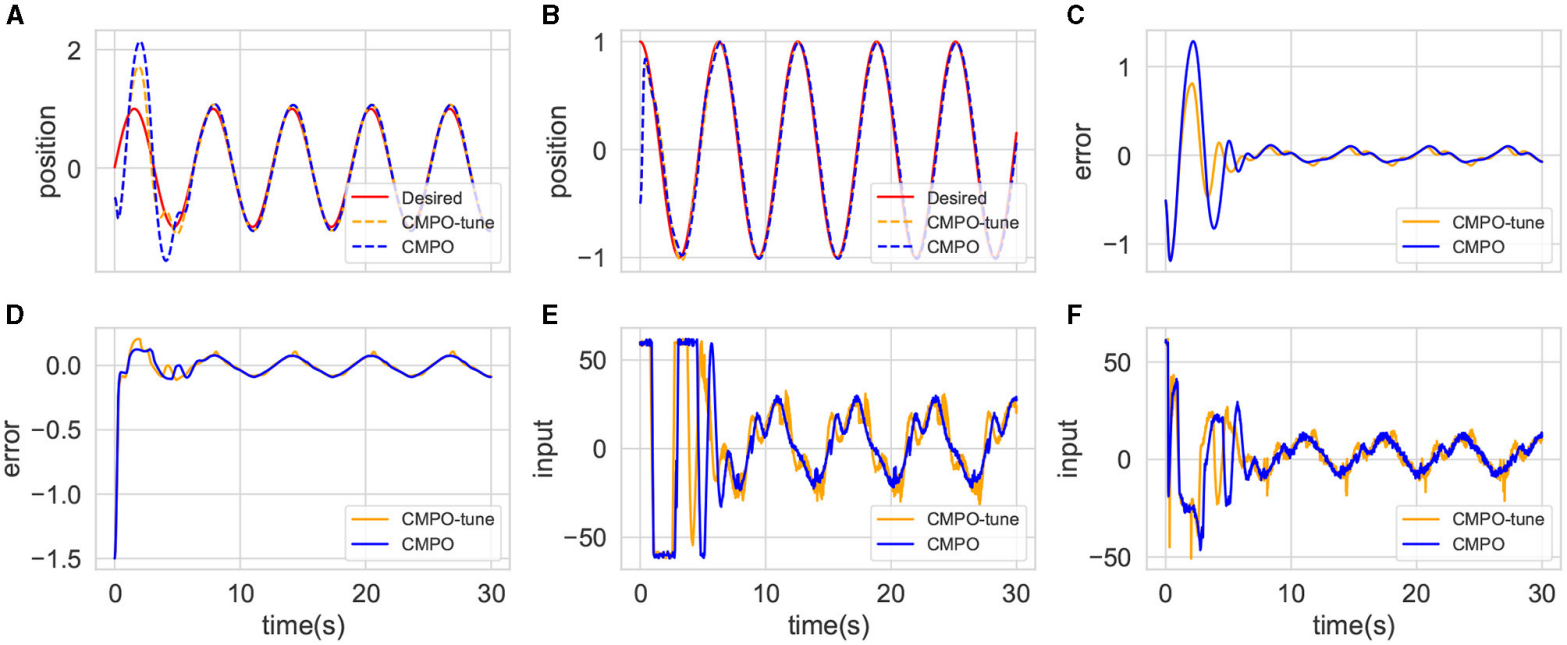
Comparison of tracking performance between CMPO and fine-tuning CMPO in the big-change environment. The display content of each component image is similar to that of Figure 4.

**Figure 7:**
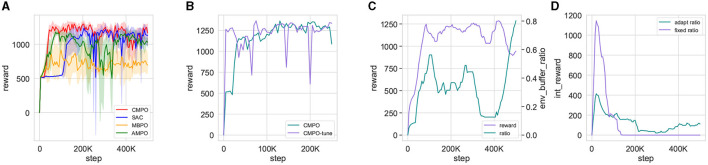
Visualization of agents training metrics in the basic environment. **(A)** is the reward curve for CMPO compared with baseline RL, obtained by taking the mean and standard deviation of five times training in the basic environment; **(B)** compares the reward curves trained in the basic environment with that from fine-tuning the model in big change; **(C)** is a visualization of the basic environmental reward curve vs. the adaptive environment buffer ratio; **(D)** visualizes the agents using fixed and adaptive ratios to output intrinsic rewards in the basic environment.

Finally, a CMPO with the same parameters is simulated in the big-change environment, which is then compared with the fine-tuned CMPO. The tracking results are shown in Figure 6. The original CMPO takes longer to converge in performance, which is shown in Figure 6A. Based on the data presented in Figure 6E, it is evident that the cause of the issue lies in the input saturation being more severe. Fine-tuning CMPO requires only approximately 20% of the original training cost, as shown in Figure 7B, demonstrating the value of generalization.

#### 7.2.2 Training performance

In this experiment, the CMPO is performed with other RL algorithms, including SAC, MBPO, and AMPO in the basic environment. The detailed parameter settings are shown in Appendix B.

**Figure 8:**
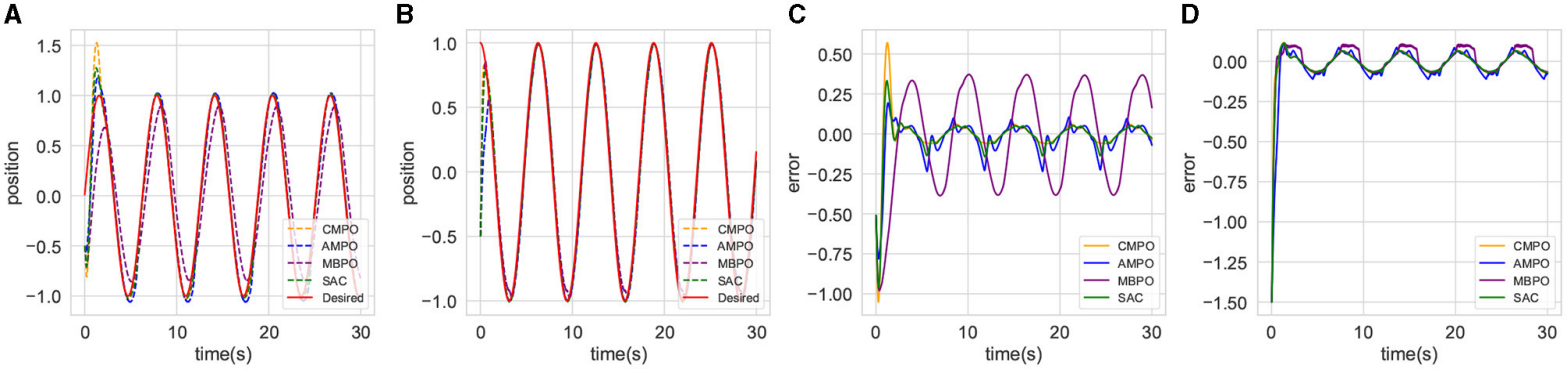
Comparison of the tracking effectiveness of CMPO with other baseline RL in the basic environment. **(A, B)** represent the curves of the position tracking over time for joint 0 and joint 1; **(C, D)** respectively represent the curves of the tracking error over time for joint 0 and joint 1.

The reward curves are shown in Figure 7A. It can be observed that CMPO trains faster than all baselines in the basic environment, indicating that the curiosity model plays an important role in enhancing sampling efficiency and robustness. In addition, the trajectory tracking performance of the algorithms is compared and can be accessed in Figure 8. It is found that CMPO tracking outperforms all the baseline algorithms while achieving asymptotic performance slightly better than that of SAC.

To further validate the effect of the ratios, the simultaneous change in the ratios with the rewards is shown in Figures 7C, D. In each episode of agent training, Figure 7C shows the variation of the environment buffer sampling ratio with rewards. The ratios reflect a general upward trend throughout training. But in detail, a trend of decreasing rewards is repeatedly predicted to elevate the ratios, which is consistent with the theoretical remarks (Lai et al., 2021). The intrinsic reward for curiosity with a fixed ratio has much larger outputs than the adaptive ratio at the beginning, as shown in Figure 7D. In addition, the adaptive ratio still maintains an effective curiosity reward output in the later stages.

**Figure 9:**
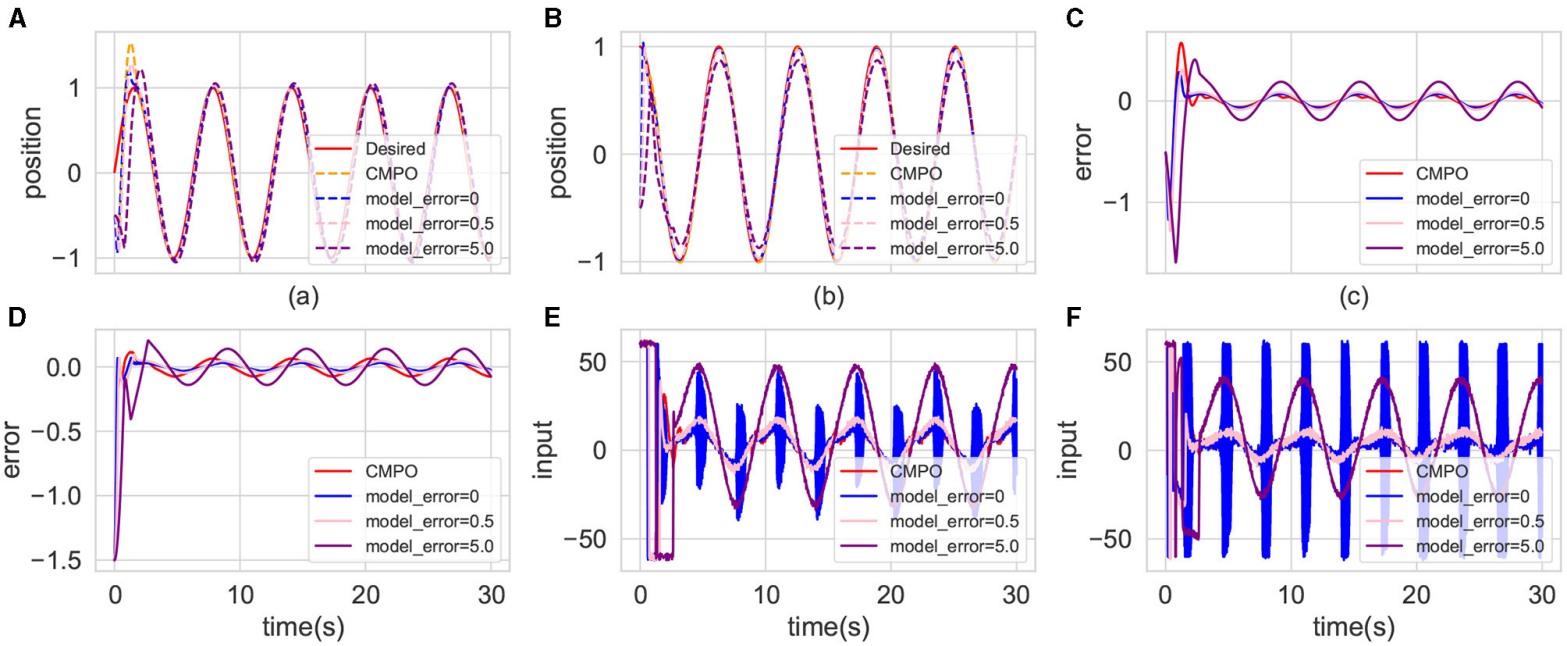
Comparison of the tracking effectiveness of CMPO in the same parameters with cutting-edge controller in the basic environment. The display content of each component image is similar to that of Figure 4.

Figure 9 illustrates the control performance of the advanced controller with different robotic model errors and the CMPO in the basic environment. The advanced controller demonstrates optimal control performance in error-free conditions, as shown in Figures 9A–D. However, as shown in Figures 9E, F, the advanced controller exhibits the highest input fluctuation and associated input cost at this juncture. Conversely, the CMPO approach achieves comparable control performance while minimizing input costs. Due to the advanced controller's high sensitivity to variations in the robotic model, its control performance diminishes with increasing model error, ultimately falling behind that of the CMPO algorithm.

**Figure 10:**
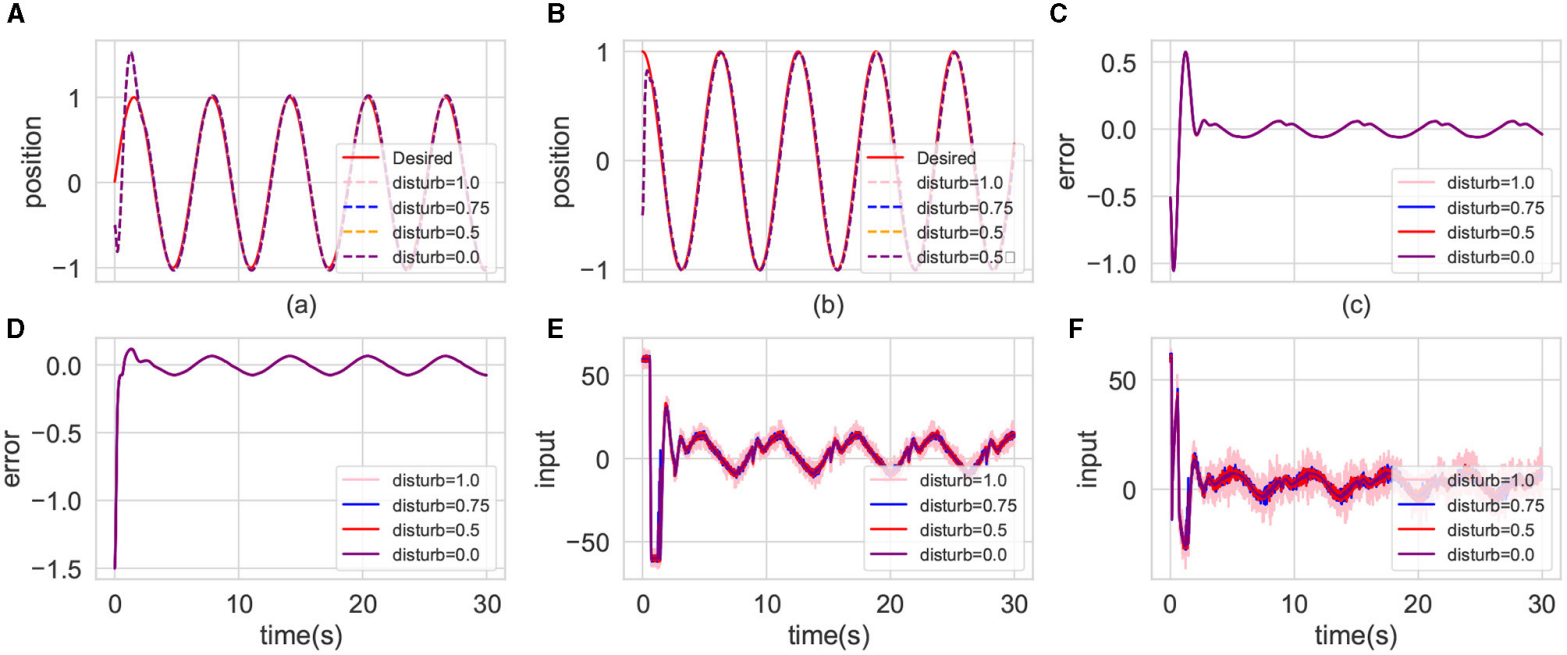
Comparison of the tracking effectiveness of CMPO in the same parameters with different input disturbance frequency in the basic environment. The display content of each component image is similar to that of Figure 4.

In uncertain environments, low-frequency or high-frequency disturbance inputs can reveal controller's immunity to interference. Figure 10 depicts the control performance of the CMPO algorithm under varying disturbance probabilities. Figures 10A–D demonstrate that disturbances within bounded ranges exhibit negligible impact on control performance, irrespective of their frequency characteristics. Notably, Figures 10E, F reveal discernible differences in moment inputs generated under differing disturbance scenarios. Specifically, with increasing disturbance probability, greater input magnitudes are employed to mitigate the disturbance effects.

#### 7.2.3 Ablation experiment

**Figure 11:**
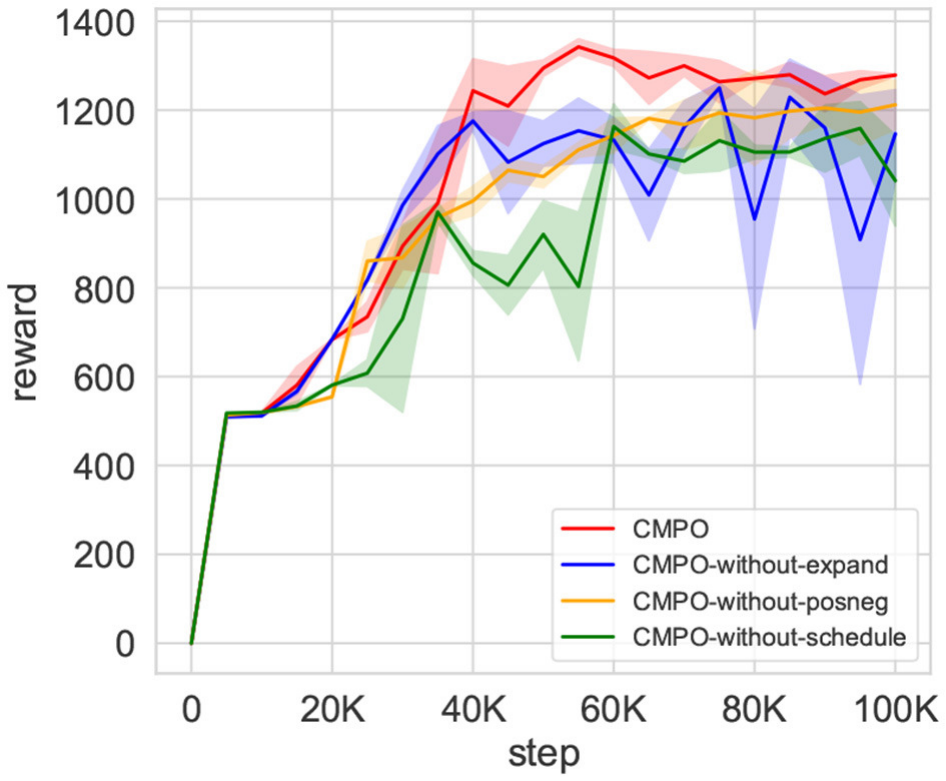
The results of the ablation experiment of CMPO. After removing the individual modules of CMPO separately, the training is performed in the basic environment with a step size of 100K. Comparison of ablated modules includes curiosity expansion, positive-negative curiosity, and adaptive scheduling buffer ratio.

To demonstrate the effect of model enhancement across modules, an ablation experiment is performed. The result is shown in Figure 11. Reward convergence is less stable when curiosity expansion is no longer applied, although the reward ascends slightly faster than before. The rate of convergence of rewards is substantially reduced if positive–negative intrinsic is removed. It also causes the same distress when the buffer scheduler is hidden. Moreover, the convergence results deteriorated. This experiment demonstrates the beneficial effects of all three modules in CMPO, improving the sampling efficiency of the model.

#### 7.2.4 Hyperparametric sensitivity experiment

**Figure 12:**
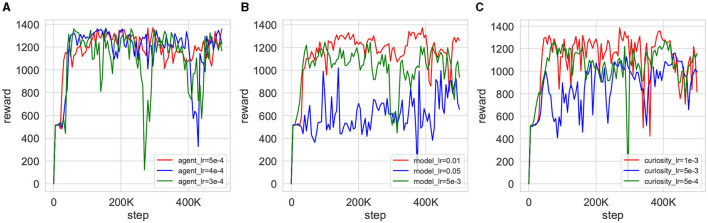
Training reward curves for the CMPO parameter sensitivity experiment. Reward changes for agent training are obtained by increasing and decreasing the learning rate of the current gates and after training once in the base environment with a step size of 500K. **(A)** demonstrates the effect of different learning rates of the agent on training, **(B)** exhibits the impact of different learning rates of the world model on training, and **(C)** compares the influence of different learning rates of the curiosity network on training.

Parameter sensitivity experiments offer valuable insights into the impact of parameter variations on the efficacy of model training. Figure 12 shows the simultaneous testing of the agent learning rate λ_*a*_, λ_*Q*_, λ_α_ (denoted as agent_lr), the curiosity network learning rate λ_icm_ (denoted as curiosity_lr), and the world model learning rate λ_θ_ (denoted as model_lr). As shown in Figure 12A, it is evident that the agent's performance shows minimal sensitivity to changes in the learning rate parameter. Despite fluctuations in the reward curve corresponding to different parameters, the overall trend toward eventual convergence remains consistent. Conversely, Figures 12B, C illustrate that both the world model and curiosity network exhibit sensitivity to changes in the learning rate. Particularly in the world model shown in Figure 12B, excessively small learning rates can lead to failure in achieving convergence during agent training.

## 8 Conclusion

This study investigates agent-efficient sampling and training for robot manipulators with input saturation in uncertain environments. The combination of the curiosity model and a traditional model-free controller is developed to strengthen trajectory tracking performance. Specifically, a notion of positive–negative intrinsic is defined and used in conjunction with adaptive ratios. The gain policies implemented by the agent based on CMPO are empirically concluded to effectively potentiate control performance. In addition, the framework can achieve low-cost fine-tuning to boost tracking capabilities in different scenarios, which facilitates the application. By virtue of these experimental results, augmented model sampling efficiency and competitive control performance are exhibited.

The aforementioned procedure is executed via numerical simulations. In forthcoming advancements, experimental endeavors will leverage robotic manipulators equipped with expanded input–output capacities and augmented degrees of freedom. Correspondingly, the creation of increasingly intricate application environments is envisaged.

## Data availability statement

The raw data supporting the conclusions of this article will be made available by the authors, without undue reservation.

## Author contributions

TW: Conceptualization, Data curation, Investigation, Methodology, Software, Validation, Visualization, Writing – original draft, Writing – review & editing. FW: Formal analysis, Funding acquisition, Investigation, Methodology, Project administration, Resources, Supervision, Writing – original draft, Writing – review & editing. ZX: Methodology, Project administration, Resources, Supervision, Writing – review & editing. FQ: Methodology, Project administration, Resources, Supervision, Writing – review & editing.
