# Supplementary material for: Curiosity model policy optimization for robotic manipulator tracking control with input saturation in uncertain environment
Source: Front Neurorobot. 2024 May 1;18:1376215. doi: 10.3389/fnbot.2024.1376215 (PMC11234325; doi:10.3389/fnbot.2024.1376215)
Supplement: Supplementary file 1 [file Data_Sheet_1.pdf]

Supplementary Material

1 ENVIRONMENT PARAMETERS

The detailed parameters of the two-link (2-DOF) manipulator in different environments can be found in Table S1.

Symbol	Parameter	basic env	small-change env	big-change env
q_1	first link position	$0 \sim 2\pi$ rad	$0 \sim 2\pi$ rad	$0 \sim 2\pi$ rad
q_2	second link position	$0 \sim 2\pi$ rad	$0 \sim 2\pi$ rad	$0 \sim 2\pi$ rad
g	gravity acceleration	9.8	9.8	9.8
r_1(m)	first link length	1.0	0.9	2.0
r_2(m)	second link length	1.0	0.6	2.0
m_1(kg)	first link mass	0.5	0.9	0.25
m_2(kg)	second link mass	0.5	0.6	0.25

Table S1. Symbols and parameters for robotic manripulator

The format of the parameters, inertial matrix, centrifugal and Coriolis force matrix, gravitational force effect of the robot in the environment are as follows:

$$M(\boldsymbol{q}) = \begin{bmatrix} M_{11}(q) & M_{12}(q) \\ M_{21}(q) & M_{22}(q) \end{bmatrix}, G(\boldsymbol{q}) = \begin{bmatrix} G_{11}(q) \\ G_{21}(q) \end{bmatrix}, \quad C(\boldsymbol{q}, \dot{\boldsymbol{q}}) = \begin{bmatrix} C_{11}(q) & C_{12}(q) \\ C_{21}(q) & C_{22}(q) \end{bmatrix}$$

where

$$\begin{cases} M_{11}(q) = (m_1 + m_2)r_1^2 + m_2 r_2^2 + 2m_2 r_1 r_2 \cos(q_2) \\ M_{12}(q) = m_2 r_2^2 + m_2 r_1 r_2 \cos(q_2) \\ M_{21}(q) = m_2 r_2^2 + m_2 r_1 r_2 \cos(q_2) \\ M_{22}(q) = m_2 r_2^2 \\ C_{11}(q) = -m_2 r_1 r_2 \sin(q_2)\dot{q}_2 \\ C_{12}(q) = -m_2 r_1 r_2 \sin(q_2)(q_1 + q_2) \\ C_{21}(q) = m_2 r_1 r_2 \sin(q_2)\dot{q}_1 \\ C_{22}(q) = 0 \\ G_{11}(q) = (m_1 + m_2)r_1 \cos(q_2) + m_2 r_2 \cos(q_0 + q_1) \\ G_{21}(q) = m_2 r_2 \cos(q_0 + q_1) g \end{cases}$$

2 RL PARAMETERS

The detailed parameter settings of CMPO and other basic algorithms are presented in Table S2.

Parameter	SAC	MBPO	AMPO	CMPO
obs_size	8	8	8	8
action_size	6	6	6	6
timesteps	500k	500k	500k	500k
agent_train_gap	4	4	4	4
model_train_gap	-	1.25k	2.5k	5k
model_rollout_length	-	$4 \to 40, 1 \to 50$	$4 \to 40, 1 \to 50$	$4 \to 40, 1 \to 50$
TS_frequency	-	1	1	1
model_end_adapt	-	-	12 ep/200 rollout	-
env_buffer_size	100k	100k	100k	65k
total_sample_size	-	256	512	256
env_sample_size	64	-	-	-
env_sample_ratio	-	0.3	0.3	auto
net_units	256	256	256	256
net_layers	2	2	2	2
gamma	0.99	0.99	0.99	0.99
tau	5e-3	5e-4	5e-4	5e-4
beta	-	-	-	0.5
agent_lr	5e-4	5e-4	5e-4	5e-4
model_lr	-	0.01	0.01	0.01
adapt_lr	-	-	5e-4	-
timesteps/episode	500k/100	500k/100	500k/100	500k/100

Table S2. Parameter settings for SAC, MBPO, AMPO and CMPO
